# Are Baby Boomers hazardous drinkers as they age? An exploratory interRAI study

**DOI:** 10.1177/10398562251346622

**Published:** 2025-05-30

**Authors:** Yoram Barak, Gil Rothschild-Eliasi, Paul Glue, Robin Turner

**Affiliations:** Department of Psychological Medicine, Dunedin School of Medicine, University of Otago, Dunedin, New Zealand; Faculty of Law, Haifa University, Haifa, Israel; Department of Psychological Medicine, Dunedin School of Medicine, University of Otago, Dunedin, New Zealand; Biostatistics Centre, Department of Preventive and Social Medicine, University of Otago, Dunedin, New Zealand

**Keywords:** interRAI, alcohol use, older adults, Baby Boomers

## Abstract

**Objective:**

Adults born between 1946 and 1964 (‘Baby Boomers’; BBs) reportedly show an increase in alcohol use creating a critical focus for prevention. We studied age-specific alcohol use patterns in a national dataset.

**Methods:**

New Zealanders 65 years and older who completed an international resident assessment instrument (interRAI-HC) interview were included.

**Findings:**

Data from 166,524 participants was analysed (mean age, 82.3 ± 7.8 years; 100,315 (60.2%) females). Of these 14,382 were BBs (mean age, 67.8 ± 2.3 years; 7581 (52.7%) females). Alcohol use declined with age. The majority of interviewees did not use any alcohol in the last 14 days. Highest number of drinks in the last 14 days were significantly higher in BBs (Chi^2^ = 647; DF = 3; *p* < .001). Hazardous drinking, defined as having had five or more drinks in any ‘one sitting’, was 3 times more frequent in BBs (2.99% vs 1.0%; *p* < .001). However, when fitting a logistic regression model to capture enough events, for those aged 68 to 74 this effect was reduced. Women had lower adjusted odds of hazardous drinking.

**Conclusions:**

Some BBs exhibit higher rates of hazardous alcohol use than older adults – the ‘Silent Generation’. This calls for policy makers to raise awareness and offer prevention – especially to younger BBs.

## Public health significance

### Evidence before this study

Historically, there has been one chronic medical condition that declined with increasing age, substance use. However, unlike previously ageing populations Baby Boomers (BBs) high use of illicit substances in earlier life is notable.

A Comprehensive PubMed and PsycINFO search using the Mesh terms ‘baby boomers’ and ‘alcohol’ identified articles wherein BB alcohol use was reported. The published literature emphasizes that substance use, including alcohol, remains high as this population transitions into older adulthood, thus making substance use in the elderly the fastest growing health problems in high-income countries.

### Added value of this study

Problem drinking is an important issue in the elderly population. Even though drinking declines with age the rate of this decline, at least in the US, is slowing. The present study examined the alcohol use of older adults reported in interRAI assessments undertaken in a large national sample. Younger BBs were found to be more hazardous drinkers than the previous generation of older adults were, at a similar age. This finding is novel and a focus on hazardous drinking is a unique advantage of our research.

### Implications of all the available evidence

It is not clear why a minority of older adults are drinking hazardously. Health Promotion Agencies recommend at least two alcohol-free days a week for everyone which is even more relevant to older adults. The present analysis demonstrates hazardous drinking patterns in BBs and needs to serve as a call for further collaborative efforts between anthropologists, medical professionals, legislators, and epidemiologists into the domain of alcohol use in older adults.

## Introduction

In many of today’s societies, alcoholic beverages are a routine part of the social landscape.^1^ Historically, there has been one chronic medical condition that declined with increasing age, substance use. However, unlike previously ageing populations Baby Boomers (BBs) high use of illicit substances in earlier life is notable.^2^ Additionally, it has been observed that substance use, including alcohol, remains high as this population transitions into older adulthood, thus making substance use in the elderly one of the fastest growing health problems in the US.^3^ There are several theories that may explain the emergence or continuation of substance use disorders in later life. These include increasing older adult populations secondary to increased life expectancy which allows populations to use substances for longer or it may be secondary to attitudes surrounding drug use, which are unique to the BBs. Older adults may begin to use alcohol as a part of their social life, fun, and enjoyment. They also tend to adopt the drinking habits of those close to them who view it as a social norm. Older adults tend to drink to deal with life’s difficulties such as anxiety; loneliness; loss of partners, family, friends; or loss of physical activity and mobility.^4,5^

Recent surveys have emphasized concerns about the rising alcohol-related morbidity and mortality in the United States.^6^ Meta-analysis-derived estimates of average annual percentage increase in the prevalence of alcohol use were 0.30% per year. Consistency in the degree to which trends have affected various demographic groups was noted with the changes in prevalence for alcohol use shown to be large and positive for ages 50 to 64 and 65 and up.^7^

Similar trends are recorded in Europe, the UK, and Australia. In the Netherlands Mental Health Survey, the past-year prevalence of heavy alcohol use was higher in people older than 55 years compared to younger people. Heavy alcohol use was associated with higher level of education in older adults compared to younger adults.^8^ Roche and Kostadinov reported that rising prevalence of risky drinking in Australia cannot be attributed solely to increasing numbers of older people corresponding to an additional 400 000 people drinking at potentially problematic levels.^9^ They also speculated that BBs may be important contributors to the changing pattern of alcohol consumption. In the UK, alcohol problems in older adults aged 65 years or over have risen steadily over the past decade with the number of people aged over 65 years requiring treatment for a substance use problem, more than doubling between 2001 and 2020.^10^

Despite growing use and treatment seeking among older adults, a paucity of published or funded work exists in the area of BBs alcohol use.^11^ In New Zealand, a 2015 study raised concerns that BBs drank more hazardously than people from older cohorts.^12^ This was echoed by a publication by Towers and colleagues in 2019 and by Szabo and colleagues in 2021.^13,14^ Both studies used the Alcohol Use Disorders Identification Test-Consumption (AUDIT-C) to identify drinking hazardously.^13^ Survey data combined with retrospective life course history interviews were collected from 749 non-lifetime alcohol abstainer adults aged 61–81 years. Latent class growth analysis yielded two life course trajectories for women while men showed three trajectories but, in both genders, there was a decrease in alcohol use with ageing yet a minority of older adults remained hazardous drinkers.^14^

The aim of the present analysis was to evaluate alcohol use patterns in a large national dataset of older adults in order to establish whether BBs are indeed using alcohol more hazardously than previous cohorts of ageing adults.

## Methods

The interRAI is an organization that develops and validates assessment instruments for vulnerable populations. It offers comprehensive assessment that has been widely used as a validated resource for studying demographic, behavioural, and health-related outcomes in older adults in New Zealand.^15–17^ The interRAI – Home Care (interRAI-HC) version is a 236-item electronically recorded assessment that includes physical, psychological, and cognitive domains. In New Zealand, an interRAI-HC is mandatory in the community as an assessment for required services for older people or prior to long-term care facility placement.^18^

New Zealanders 65 years and older who completed their first interRAI-HC assessment during the study period and had consented for their data to be used for research were included in this study. If participants underwent multiple assessments during the study period, only data from the initial assessment were reviewed. Participant data were anonymized; no personal identifying data, such as National Health Index number or date of birth, were accessible.^19^ In the present study, all interRAI-HC assessments undertaken between 2012 and 2019 in New Zealand (NZ) served as the analysed dataset.

### Measurements

The interRAI assessment variables analysed in the present study included demographic details and alcohol use and patterns of use as captured by the interRAI section ‘J – Health Conditions’. Alcohol use is assessed by the interRAI as follows: ‘Highest number of drinks in any “single sitting” in the last 14 days. 0 = none, 1 = 1, 2 = 2–4, and 3 = 5 or more’.

### Hazardous Drinking

In the present study, the definition of ‘Hazardous Drinking’ corresponds to the Alcohol Use Disorders Identification Test (AUDIT) definition. The AUDIT is the most widely tested instrument for screening in primary health care.^20^ The AUDIT provides optimal screening thresholds for alcohol use among men and women and has been shown to detect hazardous levels of drinking amongst those older than 65 when compared to younger groups.^21,22^ The AUDIT’s assessment of the standard threshold for hazardous drinking has been updated as Dawson and colleagues^23^ found that a threshold of five or more drinks in one sitting best identified hazardous drinking in older drinkers irrespective of gender in the general population and this was confirmed by Aalto and colleagues.^24^ These definitions are also in line with a recent systematic review of self-report measures used in epidemiological studies to assess alcohol consumption among older adults.^25^ However, the absence of data on quantity and frequency reduces the accuracy of the definition herein used.

### Statistical analysis

Participants’ characteristics were described using mean and standard deviation for age and number and percent for gender, generation, and age when categorized. Missing data were minimal (<3%) and therefore excluded as they would have little impact on the results. Logistic regression was used to estimate odds ratios for the primary outcome of hazardous drinking with age, gender, and generation all included categorically in the model. Unadjusted estimates were also obtained by including each variable in the model separately. A likelihood ratio test was used to test the significance of a potential interaction between age and generation. Categorical age was also fitted in the model continuously to estimate the linear trend across the ordered categories. Statistical significance was determined by *t* test (two-sided when appropriate) *p* < .05. A sensitivity analysis was conducted excluding those aged 74 years from the logistic model due to the sparse data.

### Compliance with ethical standards

Ethical approval for the proposed survey was obtained from the University of Otago Ethics Committee and the Department of Psychological Medicine Ethics Committee, approval # HD17/064.

Data will be made available upon request.

### Results

In the present analysis, we utilized 166,524 interRAI-HC first assessments undertaken in New Zealand during the period July 2013 to June 2020. This cohort is thus composed of 13.2% of the population 65 years and older in the country.^26^

### Participants

Baby Boomers are defined as persons born between 1946 and 1964,^27^ while persons born before 1946 are named as the ‘Silent Generation’ in geriatric epidemiology.^28^ In the present dataset, there was a majority (152,146; 91.4%) of older adults of the Silent Generation (mean age, 83.7 ± 6.7 years; 92,737 (61.0%) females) and there were 14,378 (8.6%) Baby Boomers (mean age, 67.8 ± 2.3 years; 7578 (52.7%) females).

### Alcohol use

The great majority of assessments – 135,137 (81.2%) – recorded interviewees reporting not using alcohol in any single sitting in the last 14 days. This held true for both females (85.4% not using alcohol) and males (74.7% not using alcohol). In the Silent Generation 85.4% of females and 74.5% of males did not use alcohol in any sitting in the last 14 days. Similar rates were found for the BBs: 85.4% for BBs females and 76.1% for BBs males (see Table 1 for details).

**Table 1.** Drinking by gender and generation

Drinking category	Overall			Silent Generation			Baby Boomers		
	Female	Male	Total	Female	Male	Total	Female	Male	Total
0	85,682 (85.4)	49,455 (74.7)	135,137 (81.2)	79,211 (85.4)	44,277 (74.5)	123,488 (81.2)	6471 (85.4)	5178 (76.1)	11,649 (81.0)
1	10,716 (10.7)	9634 (14.6)	20,350 (12.2)	10,086 (10.9)	8978 (15.1)	19,064 (12.5)	630 (8.3)	656 (9.6)	1286 (8.9)
2	3305 (3.3)	5759 (8.7)	9064 (5.4)	2939 (3.2)	5111 (8.6)	8050 (5.3)	366 (4.8)	648 (9.5)	1014 (7.1)
3	612 (0.6)	1361 (2.1)	1973 (1.2)	501 (0.5)	1043 (1.8)	1544 (1.0)	111 (1.5)	318 (4.7)	429 (3.0)
Total	100,315	66,209	166,524	92,737	59,409	152,146	7578	6,800	14,378

Drinking category: Highest number of drinks in any ‘single sitting’ in the last 14 days. 0 = none, 1 = 1, 2 = 2–4, and 3 = 5 or more.

interRAI assessments demonstrated significant differences between the BBs and the Silent Generation on alcohol use patterns (Chi^2^ = 647; DF = 3; *p* < .001) with BBs having higher rates in the two higher drinking categories. Hazardous drinking, defined as having had four or more drinks in any ‘one sitting’, was 3 times more frequent in BBs (3.0% vs 1.0%; Odds Ratio = 3.0, 95% confidence interval 2.7 to 3.3, *p* < .001). The odds ratio for female hazardous drinking compared to male hazardous drinking was 0.30 95% CI 0.24 to 0.38, *p* < .001 for BBs and OR = 0.30, 95% CI 0.27 to 0.34, *p* < .001 for the Silent Generation (see Table 2 for details of hazardous drinking).

**Table 2.** Age and generation hazardous drinking

Age	Silent Generation		Baby Boomers		Total
	Number hazardous drinking (%)	Number in age group	Number hazardous drinking (%)	Number in age group	
65	0 (0.0)	0	89 (3.0)	2963	2963
66	0 (0.0)	0	65 (3.1)	2131	2131
67	0 (0.0)	51	59 (2.8)	2122	2173
68	14 (2.9)	478	53 (2.9)	1844	2322
69	26 (2.7)	954	51 (3.1)	1626	2580
70	40 (2.8)	1410	38 (2.8)	1360	2770
71	52 (2.6)	2010	39 (3.5)	1127	3137
72	43 (1.6)	2730	25 (3.1)	794	3524
73	76 (2.2)	3479	7 (1.9)	364	3843
74	80 (1.9)	4185	3 (6.5)	46	4231
75+	89 (1.9)	4649	0 (0.0)	1	136,850
Total	1544 (1.0)	152,146	429 (3.0)	14,378	166,524

Hazardous drinking = 4 or more drinks in one sitting best identifies hazardous drinking in older drinkers.

In order to fit a logistic regression model, there needed to be sufficient people within category and with enough events to fit the model. Due to the different generations having different ages of assessment, we could only model those aged 68 to 74 years where we had both BB and the Silent Generation and enough events of hazardous drinking. There was no evidence of an interaction between age and generation (*p* = .213), that is, the pattern of hazardous drinking by age is similar for both BB and the Silent Generation. The unadjusted and adjusted logistic regression results are shown in Table 3.

**Table 3.** Logistic regression results for those aged 68 to 74 years old

Characteristics	Unadjusted		Adjusted	
	OR	*p*-Value	OR	*p*-Value
Generation				
BB	1.40 (1.18, 1.67)	<0.001	1.22 (0.99, 1.50)	0.066
Silent Generation (reference group)				
Gender				
Female	0.32 (0.26, 0.38)	<0.001	0.32 (0.26, 0.38)	<0.001
Male (reference group)				
Age in years				
68 (reference group)				
69	1.04 (0.74, 1.44)	0.838	1.04 (0.74, 1.45)	0.823
70	0.98 (0.70, 1.36)	0.882	1.02 (0.73, 1.42)	0.925
71	1.01 (0.73, 1.38)	0.973	1.09 (0.78, 1.52)	0.603
72	0.66 (0.47, 0.93)	0.018	0.75 (0.52, 1.08)	0.117
73	0.74 (0.54, 1.03)	0.074	0.86 (0.60, 1.23)	0.399
74	0.67 (0.49, 0.93)	0.017	0.79 (0.55, 1.15)	0.217

Although BBs had higher odds of hazardous drinking, after adjustment for age and gender this effect reduced and there was weak evidence of an increase in odds. The model-based unadjusted OR is lower than that reported in the previous paragraph because we had to remove those aged 65 to 67 (in order to fit the model without zero cells) where there are high rates of hazardous drinking in BBs. Women had lower odds of hazardous drinking, and this remained similar after adjustment for generation and age. Hazardous drinking tended to decrease a small amount with age, though there was weak evidence of a linear trend (*p* = .056).

Appendix Table A1 shows the impact of removing those aged 74 years or more where data was sparse. Broadly the results are similar with odds ratios slightly closer to the null and *p*-values larger with the decrease in sample size.

## Discussion

Research findings suggest that older adult BBs may retain unique alcohol use patterns.^7,29^ Results from logistic regression analyses suggest that predictors of alcohol use disorders evolve over time as BBs continue to age.^30^ The present study examined the alcohol use of older adults reported in interRAI assessments undertaken in a large national sample. The pattern of hazardous drinking did not seem to change between generations of older adults. It is of note that young BBs were found to be more hazardous drinkers than the previous generation of older adults, the Silent Generation.

Problem drinking is an important issue in the elderly population. Even though drinking declines with age the rate of this decline, at least in the US, is slowing^31^ or according to recent surveys binge drinking may even be increasing.^32^ Aggregated data from a nationally representative sample of adults aged ≥50 from the 2015 to 2019 National Survey on Drug Use and Health demonstrates that the sharpest increase in past-month binge drinking was among adults aged ≥65, with a 450% relative increase.^32^ The present study of vulnerable older adults may serve to further emphasize the deleterious effects of alcohol consumption such as 20%–30% higher all-cause mortality and cancer-specific mortality.^33^

Studies supporting the findings of the present analysis abound. In New Zealand, a recent retrospective cohort study concluded that differences in dementia prevention potential between ethnic groups in NZ are contributed to by both differential prevalence and risk factor effects including, amongst other factors, alcohol use. Public health strategies must be tailored for the ethnic populations at most risk.^34^ Significant increases in the prevalence of alcohol use and of binge drinking over the past 10 to 15 years were observed in a meta-analysis of six national surveys undertaken in the US, with emphasis that the increase in binge drinking among middle-aged and older adults is substantial and may be driving increasing rates of alcohol-related morbidity and mortality.^7^ Due to the large population size and high substance use rate of the BBs, the number of adults aged 50 or older with substance use disorder is projected to double from to 5.7 million in 2020 in the US.^29^ Similar trends are reported from Holland, Germany, Australia, and Russia.^8,9,35,36^

An attempt to create a unified anthropological theory of alcohol use sheds light on the drinking patterns of ageing BBs. The theory a priori supposes that consumption of ingested substances is inherently social and revealing of cultural processes, concerns, and symbols. This permits exploration of user behaviour and social identity in a very broad context, avoids reification of substances, and places any ‘problem focus’ into a perspective of power.^28,37^ The second element of this unified theory of ingested substances is that consumption is inherently social. ‘…consumption is eminently social, relational, and active rather than private, atomic or passive’.^38^ The premise that substances are inherently social stands in stark opposition with the underlying assumptions of much of the research in the alcohol field. The way we drink, the type of alcohol used, the people we drink with, the people we exclude from these drinking circles, the places in which we consume alcohol, the times that we do it, the people we invite to meals, and the people we exclude are all signs of an individual’s social position which is determined by factors far beyond a glass of wine.^39^ These conceptualizations help to better understand the alcohol use patterns of BBs. A quote by Dr Tony Rao, a consultant old-age psychiatrist and the author of the novel ‘Catch Me When I Fall’ is insightful: ‘…as a BB, I often ask myself why so few older people like me are willing to become sober? The alcohol-centric environment in which we grew up is a hard culture to break free from….If we wanted to fit in – like it or not – drinking was a given…’.^40^

The present analysis has several limitations that need be noted. The interRAI assessment is mandatory in a vulnerable subpopulation of older adults and thus is not representative of the general older adults in NZ. The data extracted from the interRAI assessment about alcohol use is limited and hard to extrapolate to more commonly used instruments in the field of alcohol use research. Sociodemographic data is lacking and is important as many countries, including Aotearoa New Zealand, have socioeconomic and ethnic inequities in alcohol outlet density. Structural barriers, including racism, restrict the influence of under-resourced communities and Māori further hindering community efforts to reduce their disproportionate exposure to alcohol.^41^

Drinking patterns were self-reported. This may be further complicated by recall bias in a population of vulnerable older adults. Mediators of alcohol consumption were not examined. We were unable to rule out the possibility that younger home care clients are more hazardous drinkers. A pertinent comment on use of interRAI data in the context of alcohol use research is that the interRAI recording of alcohol use has been found to be associated with nonfatal self-harm among community-dwelling older adults as well as with hospitalization in community-dwelling older adults. This is not ideal but does carry some weight towards the relevance of this single question about maximum use of alcohol in this study.^42,43^

Older adults report drinking alcohol in response to a range of life difficulties that include anxiety and loneliness, as well as those associated with the losses of ageing such as bereavement and disability. It is not clear what drives a minority of older adults to drink hazardously. The present analysis emphasizes this pattern and needs to serve as a call for further collaborative research between anthropologists, medical professionals, jurists, and epidemiologists into the domain of alcohol use in older adults.

## Appendix

**Table A1.** Sensitivity analysis with those aged 74 or over excluded from model

Characteristics	Unadjusted		Adjusted	
	OR	p-Value	OR	p-Value
Generation				
BB	1.33 (1.10, 1.60)	0.003	1.19 (0.97, 1.47)	0.103
Silent Generation (reference group)				
Gender				
Female	0.29 (0.24, 0.36)	<0.001	0.30 (0.24, 0.37)	<0.001
Male (reference group)				
Age in years				
68 (reference group)				
69	1.04 (0.74, 1.44)	0.838	1.03 (0.74, 1.45)	0.843
70	0.98 (0.70, 1.36)	0.882	1.01 (0.72, 1.42)	0.956
71	1.01 (0.73, 1.38)	0.973	1.08 (0.78, 1.51)	0.64
72	0.66 (0.47, 0.93)	0.018	0.74 (0.51, 1.06)	0.104
73	0.74 (0.54, 1.03)	0.074	0.84 (0.59, 1.21)	0.357
